# A New Perspective for Vineyard Terroir Identity: Looking for Microbial Indicator Species by Long Read Nanopore Sequencing

**DOI:** 10.3390/microorganisms11030672

**Published:** 2023-03-06

**Authors:** Ana Cruz-Silva, Gonçalo Laureano, Marcelo Pereira, Ricardo Dias, José Moreira da Silva, Nuno Oliveira, Catarina Gouveia, Cristina Cruz, Margarida Gama-Carvalho, Fiammetta Alagna, Bernardo Duarte, Andreia Figueiredo

**Affiliations:** 1 Biosystems & Integrative Sciences Institute (BioISI), Faculdade de Ciências da Universidade de Lisboa, 1749-016 Lisboa, Portugal; 2 Grapevine Pathogen Systems Lab, BioISI Faculdade de Ciências da Universidade de Lisboa, 1749-016 Lisboa, Portugal; 3 Departamento de Biologia Vegetal, Faculdade de Ciências da Universidade de Lisboa, Campo Grande, 1749-016 Lisboa, Portugal; 4 Quinta dos Murças, Esporão Company, Covelinhas, 5050-011 Peso da Régua, Portugal; 5 NBI—Natural Business Intelligence, Regia Douro Park, 5000-033 Vila Real, Portugal; 6 cE3c—Center for Ecology, Evolution and Environmental Changes & CHANGE—Global Change and Sustainability Institute, Faculdade de Ciências da Universidade de Lisboa, Campo Grande, 1749-016 Lisboa, Portugal; 7 Departamento de Química e Bioquímica, Faculdade de Ciências da Universidade de Lisboa, Campo Grande, 1749-016 Lisboa, Portugal; 8 Energy Technologies and Renewable Sources Department, National Agency for New Technologies, Energy and Sustainable Economic Development (ENEA), Trisaia Research Centre, 75026 Rotondella, MT, Italy; 9 MARE—Marine and Environmental Sciences Centre & ARNET—Aquatic Research Infrastructure Network Associate Laboratory, Faculty of Sciences of the University of Lisbon, Campo Grande, 1749-016 Lisbon, Portugal

**Keywords:** soil metagenomic, microbiome, long-read nanopore sequencing, microbial signature, grapevine

## Abstract

Grapevine is one of the most important fruit crops worldwide, being Portugal one of the top wine producers. It is well established that wine sensory characteristics from a particular region are defined by the physiological responses of the grapevine to its environment and thus, the concept of terroir in viticulture was established. Among all the factors that contribute to terroir definition, soil microorganisms play a major role from nutrient recycling to a drastic influence on plant fitness (growth and protection) and of course wine production. Soil microbiome from four different terroirs in Quinta dos Murças vineyard was analysed through long-read Oxford Nanopore sequencing. We have developed an analytical pipeline that allows the identification of function, ecologies, and indicator species based on long read sequencing data. The Douro vineyard was used as a case study, and we were able to establish microbiome signatures of each terroir.

## 1. Introduction

Grapevine is one of the most important fruit crops worldwide. In 2021, 7.3 million hectares of the word cultivated area were dedicated to viticulture to produce table grapes and wine. Portugal is one of the top 5 producers with 194 thousand hectares of vineyards and 7.3 million of hectolitres of wine production [1].

Wine can be distinguished as branded wine and terroir wine. Branded wine is produced by blending grapes from various regions, while terroir wine is made exclusively from grapes from a specific region that comprises singular characteristics [2]. It is well established that wine sensory characteristics from a particular region are defined by the physiological responses of the grapevine to its environment and thus, the concept of terroir in viticulture was established to define the physical (e.g., climate, soil) and biological (e.g., soil microbiota, grape variety, fauna) characteristics, as well as the viticulture and oenological techniques of a particular geographical location [2]. In 2010, the International Organization of Vine and Wine published an official definition of terroir: “ *Vitivinicultural “terroir”, is a concept which refers to an area in which collective knowledge of the interactions between the identifiable physical and biological environment and applied Viti vinicultural practices develops, providing distinctive characteristics for the products originating from this area. “Terroir” includes specific soil, topography, climate, landscape characteristics and biodiversity features*”, highlighting its complexity.

Among all the factors that contribute to terroir definition, soil microorganisms play a major role. Soil microbiota are a very important aspect of the ecosystem, their contribution going beyond nutrient recycling to a drastic influence on plant fitness (growth and protection). Vineyard microbiota can greatly influence the productivity of agricultural systems forming complex and dynamic associations, which can range from mutualistic to commensal to pathogenic [3,4,5,6,7]. Several examples of this have already been described for grapevine, from plant inoculation with growth-promoting bacteria leading to increase in growth and bunch production per plant [4]; to protection against pathogens such as *Botrytis cinerea* [5,6] and *Plasmopara viticola* [6,7] with natural antagonists.

Also, microbial activity has a special influence on wine production and quality [8,9]. Studies of grape and must microbiomes highlight differences in fungal and bacterial communities of different regions [10,11]. Considering that soil is a reservoir of microbial communities in the vineyard [12], terroir-associated microbiota will certainly influence not only both grapevine plants’ ability to cope with stress and its fitness, but also ultimately terroir wine sensory characteristics. Moreover, soil could represent an important source of grapevine pathogens inoculum [13], so understanding the potential differences between their abundances in soil could help in the definition the viticulture management practices.

In this study, we aimed at defining microbiome signatures and indicator species for different terroirs based on long-read Oxford Nanopore sequencing. Quinta dos Murças, an organic vineyard located in the demarcated wine region of the Douro Valley, was chosen as a case study has it presents a unique topography and exposure, and different terroirs were previously identified.

## 2. Materials and Methods

### 2.1. Vineyard Location and Sampling

**Figure 1:**
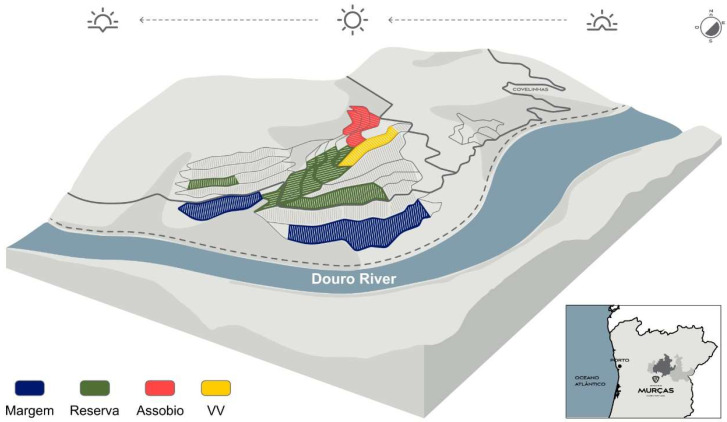
Quinta dos Murças from the wine company Esporão, located at Alto Douro Wine Region map illustration (https://www.esporao.com/pt-pt/sobre/quinta-dos-murcas/; accessed on 17 January 2023). VV—Vinhas Velhas.

Soil samples were collected at Quinta dos Murças, belonging to the wine company Esporão, located at Alto Douro Wine Region, right bank of the Douro River (41°09′11.9″ N 7°41′17.3″ W), Portugal (Figure 1). These vineyards are managed by a single owner, minimizing differences in viticulture management, and an organic viticulture approach is followed. Soil is derived from metasedimentary rocks and granitoids, being the vineyard’s soils of schistose origin. As the soil in vineyards has been strongly affected by human activities it can be classified as an anthropology [14]. Different terroirs have been defined in this vineyard, according to soil and edaphoclimatic conditions.

Four terroirs were selected for this study (Table 1), bulk soil samples were collected in 2018, during grapevine season (July and mid-September) at a depth of 5–20 cm under the canopy of adult grapevine plants (namely from the cultivar Touriga nacional—evenly represented in all the terroirs). Phytosanitary status of the vineyard was consistently monitored throughout the seasons and years. To avoid differences in soil composition, soil samples were taken between grapevine plants in the same row and fifteen sites were sampled per terroir. Soil samples were taken and mixed to obtain a homogeneous sample of about 5 kg. Homogenized soil was immediately passed through a 2-mm-pore-size sieve, and five subsamples of 100 g each were randomly selected and stored in sterile bags on dry ice at the time of sampling. Samples were then kept at −80 °C until processing.

### 2.2. Determination of Soil Physicochemical Characteristics

For each terroir, several soil parameters were evaluated, namely: pH, water content, organic matter (OM), nitrate (NO_3_^-^), total phosphate and inorganic soluble phosphate (PO_4_^−^). Five soil samples taken in mid-September were used per terroir.

A 1:10 soil water extract was prepared as described in Dias et al. [15] and used to determine soil pH by means of a selective electrode; nitrate concentration by vanadium trichloride (VCl_3_) Griess reaction [16]; and inorganic soluble phosphate (PO_4_^−^) by malachite green reaction [17]. Soil organic matter was determined by loss of ignition according to Schulte and Hopkins [18]. To determine the total soil phosphate concentration, the soil ashes obtained after ignition of the organic matter were resuspended in 1 M KCl and subsequently analysed using the malachite green reaction [17].

The concentrations of NO_3_^-^, total PO_4_^-^ and inorganic soluble PO_4_^-^ were expressed as mg or µg per gram of dry soil. Soil dry weight was obtained by drying soil samples at 45 °C until constant weight. Organic matter (OM) concentrations were also expressed as % of dry soil.

The Kruskal-Wallis test coupled with post hoc Fisher’s and a Bonferroni correction adjustment method was used to define the statistical significance of all physiological parameters between the terroirs. Statistical analysis was performed with the agricolae R package [19].

### 2.3. DNA Extraction

From each terroir soil sample, total DNA extraction was performed using the DNeasy^®^ PowerMax^®^ Soil Kit (Qiagen, MD, USA), with slight changes to the manufacturer protocol. The protocol’s input material and lysis steps were adapted to fit different soil textures (for drier soils, 5 g of input soil were used instead of 10 g and an additional heating step was performed during lysis). DNA quality and concentration were assessed by NanoDrop^TM^ One and Qubit^TM^ 4 Flourometer analysis. Three biological replicates for each terroir were obtained and used as independent samples.

### 2.4. Metagenomic Sequencing by Long-Read Nanopore

Quantification steps were performed using the dsDNA HS assay for Qubit. DNA was end-repaired (New England BioLabs, MA, USA), cleaned with Agencourt AMPure XP Beads (Beckman Coulter, High Wycombe, UK) and dA-tailed (New England BioLabs, MA, USA). The library was prepared from 1400 ng input DNA using the SQK-LSK109 kit (Oxford Nanopore Technologies, Oxford, UK) in accordance with the manufacturer’s protocol.

The library was quantified and prepared for GridION sequencing, using FLO-MIN106 flowcells, MinKNOW v18.12.4, standard 48-h run script with active channel selection enabled, until 2.5 Gb of data was collected from each sample. The mean read length of the sequenced reads was 3944 bps and the mean quality score was 10.45.

### 2.5. Bioinformatic Analysis

After removing the low-quality gDNA reads, the remaining reads were filtered for size and quality keeping reads with lengths higher than 300 bps and phred score ≥ 7, using Prinseq-lite version 0.20.4 [20].

A customized in-house analytical pipeline for long-read Nanopore sequencing was used to obtain high-accuracy taxonomical classification. The used approach had been validated through ZymoBIOMICS^TM^ Microbial Community Standard (Zymo Research Corp., Irvine, CA, USA). Taxonomic classification was performed using Kraken2 (version 2.1.2), running on default options and using a reference database including the NCBI Refseq reference genomes of Archaea, Bacteria, Viruses and the NCBI Genbank reference and representative genomes of Fungi [21].

Rarefaction curve to assess the sequencing depth (R package vegan; Figure S1) followed by a sample rarefaction to the lowest number of reads was performed (R package phylosep; 711 seed). Alpha diversity of microbiota community was assessed by calculating the Chao1 richness [22], Shannon diversity [23,24] and Pielou evenness [25] indexes using the microbiome R package [26] and compared between terroir with a Kruskal-Wallis test coupled with post hoc criterium Fisher’s least significant difference with Bonferroni correction adjustment method using agricolae R package [19]. Chao1 index considers the number of species in the community. Shannon considers the number of species and their relative abundance, measuring the uncertainty about the identity of an unknown individual. Pielou evenness index tell us if the number of individuals of each species is even or not in an area.

Taxonomical relative abundance analyses were performed using the microbiome R package [26] and compared between terroirs by Kruskal-Wallis test coupled with post hoc criterium Fisher’s least significant difference with Bonferroni correction adjustment methods [19]. Phylum relative abundance was calculated based on phylum absolute abundance and class relative abundance was calculated based on class absolute abundance. Functional analysis was performed with the microeco [27] R package using the procaryotes database FAPROTAX [28] and FungalTraits for fungi [29]. Tables S1 and S2 show the functions and ecologies associated with species.

For the identification of potential terroir-associated indicator species and functions, taxonomic reconstruction species had to meet three criteria: (1) Correlation indices analysis based on point biserial correlation coefficient must present a significant (*p* > 0.05) association to a given Terroir. This analysis was performed using the R package “indicspecies” [30], results were visualised as network generated using Cytoscape (version 3.9.1) with the edge-weighted spring-embedded layout and terroirs were defined as source nodes, the associated species as nodes and the association strength as edges. (2) Variable Importance for Projection Partial Least-Squares Discriminant Analysis (VIP-PLS-DA) must present a VIP score > 1. VIP-PLS-DA analyses were made using the R package DiscriMiner [31]. (3) relative abundance of the identified species must be significantly (*p* > 0.05) higher in the terroirs. This analysis was performed using by Kruskal-Wallis test coupled with post hoc criterium Fisher’s least significant difference with Bonferroni correction adjustment methods [19]. All R analysis were performed in R-studio version 1.4.1717.

## 3. Results

### 3.1. Physicochemical Characterization of Quinta dos Murças Terroirs

Six physicochemical characteristics (pH, organic matter content (OM), water content, inorganic soluble and total phosphate, and nitrate content) were analysed. All terroirs presented a slightly acidic pH, with Assobio and Margem covering the less acidic soils, and Vinhas Velhas the most (Table 2). Though the difference in pH is less than 1, this can greatly affect the bioavailability of nutrients [32].

Reserva shows significantly lower OM than the other terroirs. From all the terroirs analysed, Vinhas Velhas presented the highest total phosphate contents, followed by Margem. Reserva soil presented the lowest amount of total and available phosphate. Considering inorganic soluble phosphate, Margem soil presented the highest content. Nitrate was more abundant in Margem and Assobio, being significantly higher than in the Vinhas Velhas and Reserva terroirs.

### 3.2. Global Terroir Microbiome Analysis

Terroir-associated microbiome was assessed by long-read Oxford Nanopore sequencing technology. A total of 12.56 million reads were obtained. Reads with ≥300 bps and quality score ≥7 were considered for taxonomic analysis. A total of 9.19 million reads were used for taxonomic reconstruction analysis. An in-house pipeline based on k-mers taxonomic classification was used, leading to the identification of 2.73 million reads. After rarefaction (Figure S1), 9670 different microbial taxa were identified, 8558 of those at species level (Table S3). The taxa were further classified as Bacteria (5792), Viruses (313), Archaea (274) and Fungi/Oomycete (3291). Overall, 67 Phyla, 149 Classes, 351 Orders, 841 Families and 2633 Genera were identified.

Looking at the overall representation of the identified taxa, Bacteria was the most represented kingdom followed by Eukaryota (considering fungi and oomycete only) while Archaea and Virus kingdoms were the less represented (Figure S2A). Considering Phyla, seven Archaea phyla (Figure S2B) were found and of these, Euryarchaeota presented the higher relative abundances (>0.7). Candidatus Korarchaeota is the less represented phylum. Whitin Eukaryota, Ascomycota presented higher relative abundances followed by Basidiomycota, Oomycota and Mucoromycota (Figure S2C), while Sanchytriomycota presented the lower relative abundances. Considering virus, Uroviricota (virus that infect bacteria and archaea) were the more abundant, while Cossaviricota were the less abundant (Figure S2D). When looking at the Bacteria phyla (Figure S2E), Proteobacteria and Actinobacteria were the phyla with higher relative abundances, both above 0.3. The remaining phyla showed relative abundances lower than 0.1, being Balneolaeota the phylum with lowest relative abundance.

**Figure 2:**
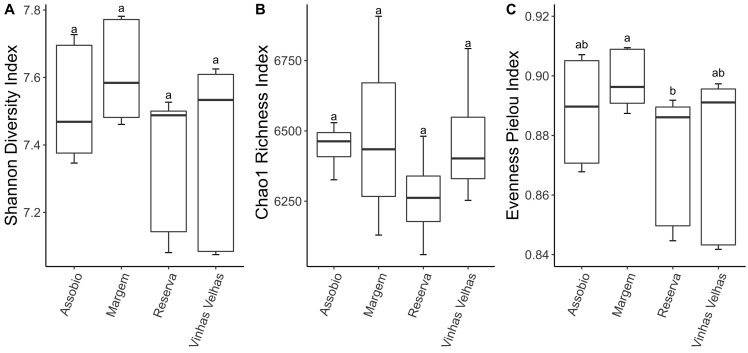
Shannon diversity index (**A**) Chao1 richness index (**B**) and Pielou evenness index (**C**). Boxplots depict N = 9 per terroir; centre line: median; lower and upper hinges: first and third quartiles (the 25th and 75th percentiles); whiskers: ×1.5 the interquartile range; letters denote significant differences at *p* < 0.05.

Alpha diversity, that determines microorganisms’ diversity within each terroir, was analysed based on the Chao1 richness, Shannon diversity and Pielou evenness indexes (Figure 2). Through both Shannon and Chao1 indexes, no significant differences were detected between the terroirs (Figure 2A,B). The Pielou evenness index, however, showed that Margem terroir presents a significant higher diversity than Reserva terroir (Figure 2C).

**Figure 3:**
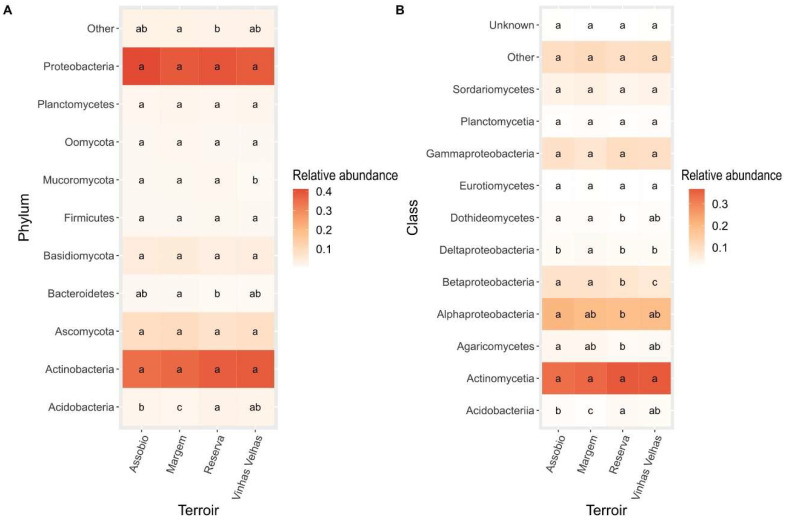
Relative abundance of Phylum (**A**) and Class (**B**) in each terroir. Letters denote significant differences at *p* < 0.05 between terroirs by Kruskal-Wallis test coupled with a post hoc Fisher’s test with a Bonferroni correction adjustment method. The taxa shown are the most abundant (detection of 0.005 for phylum and 0.01 for Class; prevalence of 0.05). The remaining taxa are included in the “Other” category. Unknowns correspond to the Class without taxonomical Classification in taxonomic ID.

Next, the most abundant phyla on the different terroirs were assessed. Within Bacteria kingdom, Proteobacteria, Plantomycetes, Firmicutes, Bacteroidetes, Actinobacteria and Acidobacteria were the more abundant phyla (Figure 3). The Bacteroidetes and Acidobacteria phyla were the only that presented statistical significances between terroirs (Figure 3A). Bacteroidetes relative abundance was significantly higher in Margem samples when compared to Reserva soil. Acidobacteria relative abundance is also significantly higher in Reserva terroir when compared to Assobio and Margem terroirs (Figure 3A). Margem is the terroir that presents the lower abundance of Acidobacteria. At class level, Deltaproteobacteria, Betaproteobacteria, Alphaproteobacteria, and Acidobacteriia abundances were significantly different between the four terroirs (Figure 3B). Alphaproteobacteria presented a higher abundance in Assobio soil when compared to Reserva soil. The terroirs Margem and Assobio presented a higher abundance of Betaproteobacteria when compared to Reserva and Vinhas Velhas. Also, the Vinhas Velhas terroir presented lowest Betaproteobacteria relative abundance. The Margem terroir showed significantly higher relative abundance of Deltaproteobacteria class when compared to all the terroirs.

Considering Eukaryota, Plantomycetes, Oomycota, Basidiomycota and Ascomycota showed no significant differences between the terroirs. Mucoromycota presents significant lower relative abundance in Vinhas Velhas soil samples when compared to the other terroirs (Figure 3A). At Class level, Eurotiomycetes and Sordariomycetes presented no significant differences between the terroirs. Dothideomycetes relative abundance is significantly higher in Assobio and Margem soils when compared to Reserva soils (Figure 3B). Agaricomycetes abundance is significantly higher in Assobio samples comparatively to Reserva samples.

### 3.3. Functional Analysis

Functional analysis was done considering the identified species, to assess metabolic or other ecologically relevant functions of the different terroir’s microbiome. Bacterial and fungal ecological functions present a very similar distribution/representation across all terroirs, globally maintaining the same relative abundances, with some subtle differences that specifically differentiate them. A cluster analysis allowed the discrimination of two groups, one including Reserva and Vinhas Velhas terroir samples and other including Assobio and Margem terroir samples, for both prokaryotic function (Figure S3) and fungi ecology (Figure S4).

**Figure 4:**
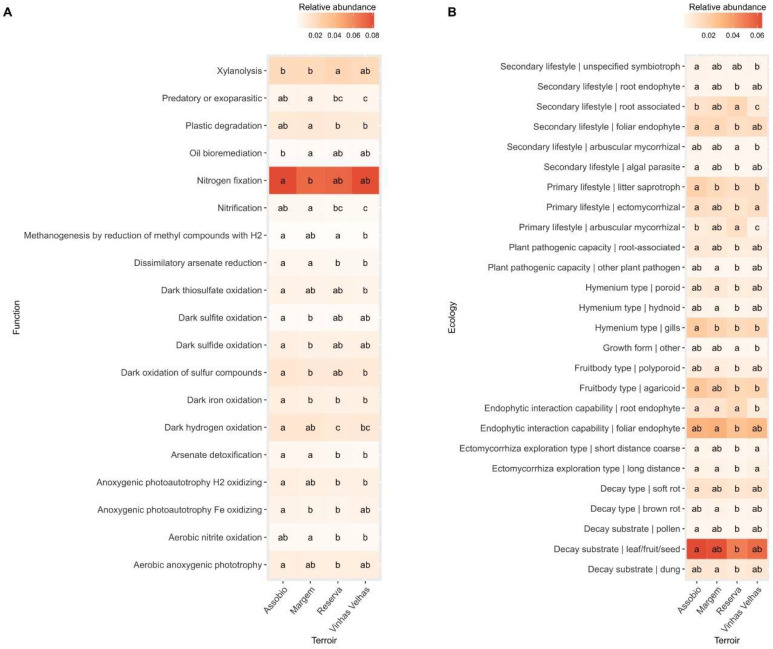
Terroir associated Bacterial functions predicted by FAPROTAX database (**A**) and Fungi ecological guilds predicted by FungalTraits database (**B**) that present statistical significance (*p* < 0.05, Kruskal-Wallis, Post hoc test Fisher’s least significant difference with Bonferroni correction adjustment methods; only functions or ecological guilds with significant differences between samples were plotted).

Although being the prevalent bacterial function identified across all terroirs, “nitrogen fixation” occurs at much higher abundances in the Assobio and Vinhas Velhas terroirs and allowed the discrimination between Assobio and Margem (Figure 4A). “Xylanolysis” appears as the second function with higher relative abundance allowing the discrimination of Reserva terroir samples from the other terroirs. Though being a less abundant function, “nitrification” also allows the discrimination between Margem and Vinhas Velhas samples.

Regarding fungi associated functions, the Reserva terroir present significant alterations in the relative contribution of “decay substrate” the function with higher relative abundance from those that present statistical significances (Figure 4B). The second higher relative abundance fungi function, “endophytic interaction capability—foliar endophyte” presented significant differences between the Margem and Reserva (Figure 4B). “Root associated” classification present the lowest abundance of in Vinhas Velhas terroir when compared to other terroirs, as well as “Endophytic interaction capability—root endophyte”. Considering mycorrhiza associated guilds, “arbuscular mycorrhizal—secondary lifestyle” and “ectomycorrhizal” present statistical differences between Reserva and Vinhas Velhas terroir samples. “Arbuscular mycorrhizal—primary lifestyle” is also significantly different in present Vinhas Velhas when comparing to the other terroirs, presenting the lowest abundance (Figure 4B). Considering “Foliar endophytes”, Margem and Reserva terroir samples were statistically different with Margem presenting a higher abundance when compared to Reserva.

### 3.4. Identification of Indicator Species and Functions for Each Terroir

**Figure 5:**
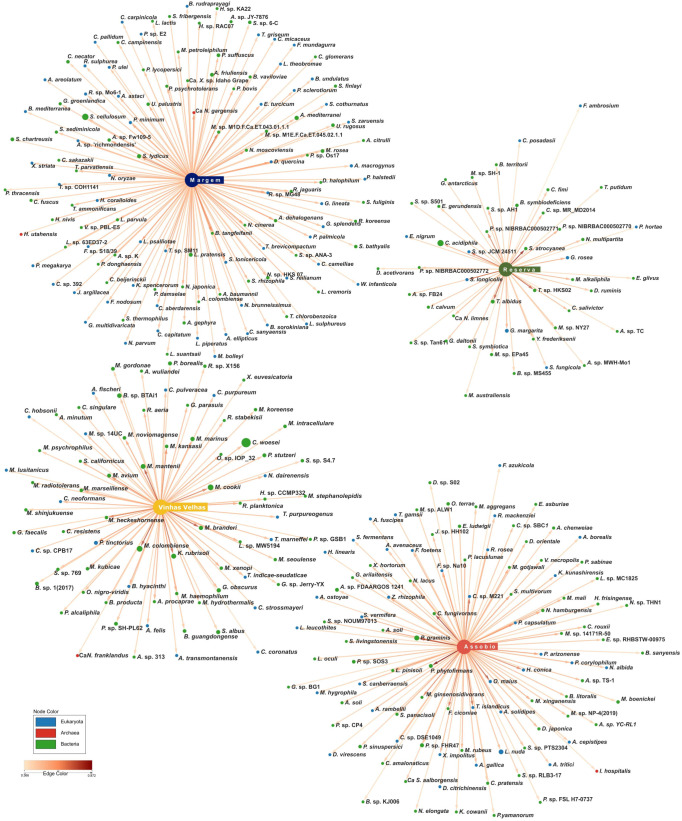
Association network of species in different Terroirs. The association network was calculated in the R package indicspecies [30]. Node size correspond to the relative abundance of the species in the terroirs, and the colour represent the kingdom that they belong to. Edge length and colour represent the strength of the association (edge-weighted, spring-embedded layout).

To depict possible indicator species responsible for terroir discrimination, a point biserial correlation analysis and a Variable Importance for Projection Partial Least-Squares Discriminant Analysis (VIP-PLS-DA) were conducted. The point biserial correlation analysis enabled the identification of 344 indicator species with significant association to the terroirs (Figure 5). Margem presented the highest number of indicator species (124), comprised in 21 different Phyla, the majority belonging to Proteobacteria (36), Ascomycota (30), Basidiomycota (18) and Actinobacteria (11). Assobio presented 100 indicator species belonging to 12 different phyla, with a predominance of Proteobacteria (36), followed by Ascomycota (22), Basidiomycota (12) and Bacteroidetes (11). Vinhas Velhas presented 75 indicator species, 35 belonging to Actinobacteria and 12 to Proteobacteria, the more represented phyla. Reserva is the terroir with the lowest number of indicator species, mostly represented in Actinobacteria (17) and Proteobacteria (12) phyla.

We were able to identify also potential indicator functions and fungal ecology for each terroir based on Prokaryotic and Eukaryotic species. Considering the prokaryotic function, Assobio is the terroirs with higher number (7), followed by Margem (3) and Reserva (1). Vinhas Velhas had no indicator function identified. Based on fungal ecology Margem was the terroirs with higher number of potential indicators (10), followed by Assobio with 4 indicator fungal ecology. Reserva and Vinhas Velhas had the same number on fungal indicator ecologies (2).

**Figure 6:**
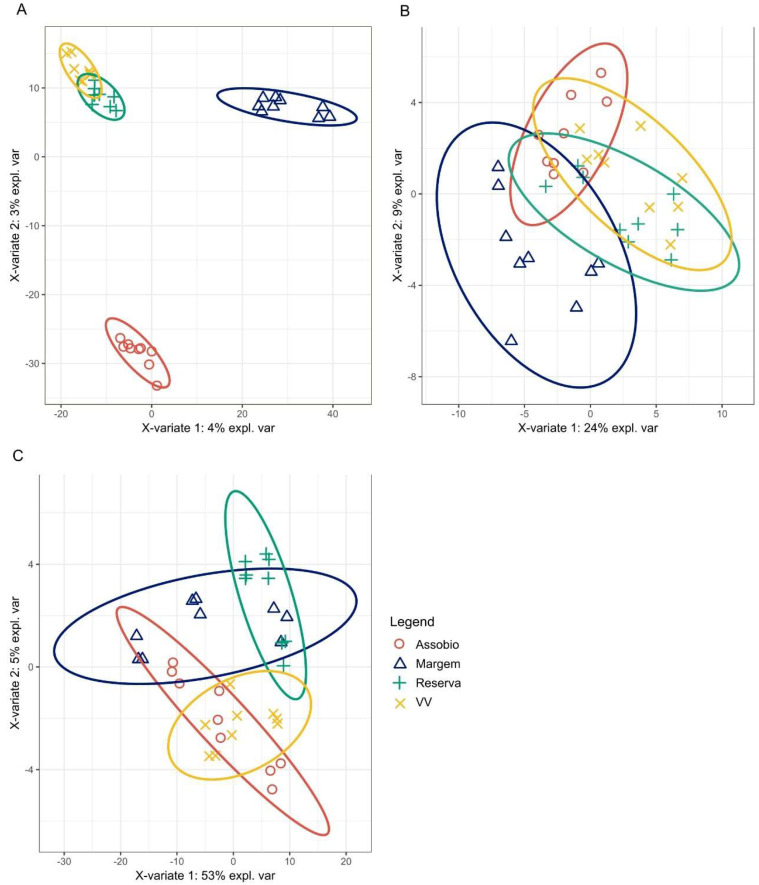
Partial least-squares discriminant analysis (PLS-DA) projection plots of the Terroir microbiome (**A**), Prokaryotic function (**B**) and Fungi ecology (**C**).

**Figure 7:**
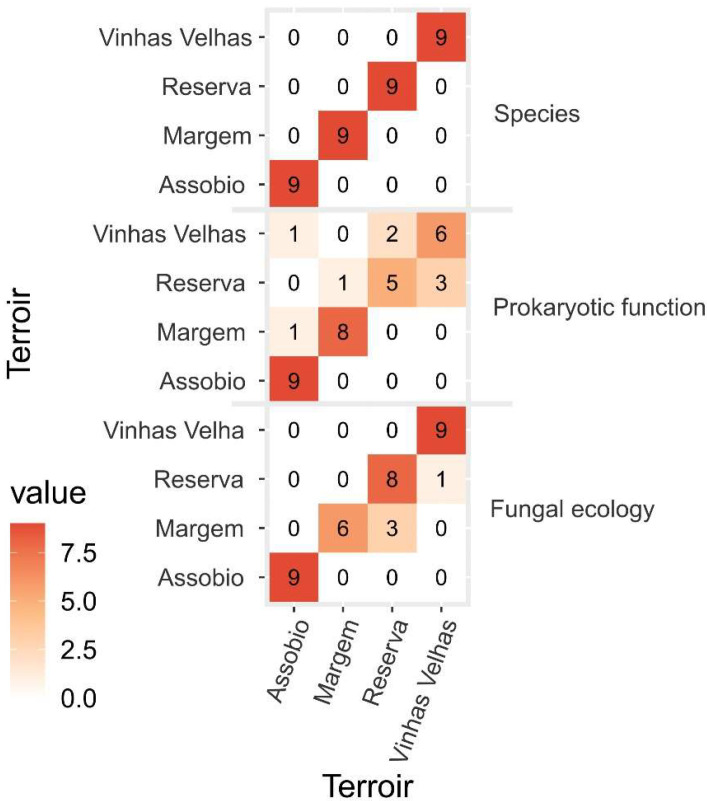
Partial Least-Squares Discriminant Analysis (PLS-DA) confusion matrix and overall model accuracy.

For visualisation purposes PLS-DA biplots were produced to better visualize the sample dispersion and grouping at microbiome (Figure 6A), prokaryotic function (Figure 6B) and fungal ecology levels (Figure 6C). Considering the terroir microbiome (Figure 6A), 3278 species presented a VIP score >1, thus contributing to PLS-DA model terroir separation. The PLS-DA biplot shows a clear spatial pattern, with the samples organized in clusters according to the collection area (terroir), resulting in 100% accuracy in correctly classifying the terroir samples according to the species abundance dataset (Figure 7). The VIP-PLS-DA projection for prokaryotic functional and fungal ecology is shown in Figure 6B,C. The PLS-DA biplot shows function organization in clusters according to the collection area (terroir), resulting in 77.78% accuracy in correctly classifying the prokaryotic functions to the terroirs and 88.89% accuracy in correctly classifying the fungal ecology indicators to the terroirs (Figure 7). Reserva and Vinhas Velhas were the terroirs with the lowest model accuracy considering indicator functions, while Margem was the terroirs with the lowest model accuracy considering fungal ecology indicators (Figure 7).

**Figure 8:**
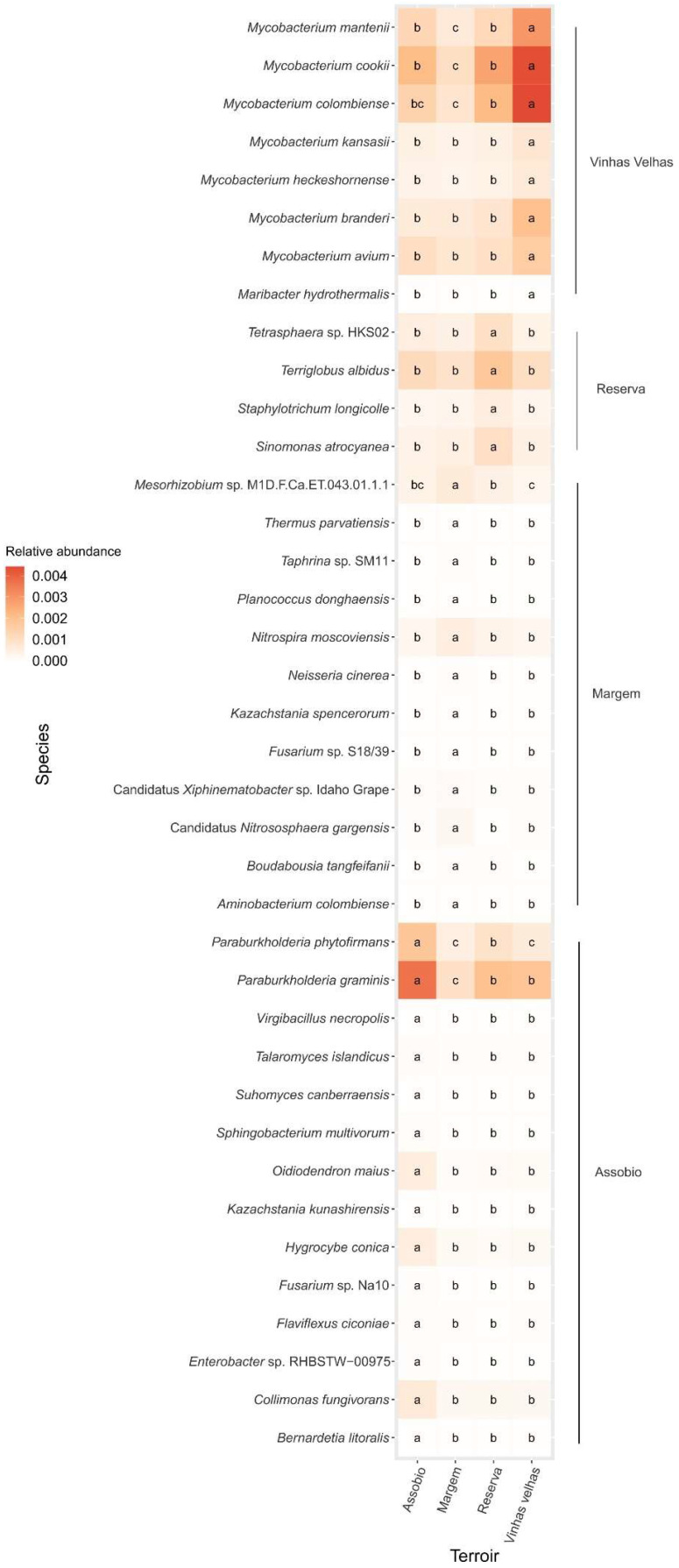
Relative abundance of terroir identified indicator species (average, N = 9). Letters denote significant differences at *p* < 0.05 between terroirs.

When comparing both approaches (VIP-PLS-DA and point biserial correlation analysis) 313 species, 11 prokaryotic function and 16 fungi ecologies were commonly identified as terroir-associated indicators. Of the species, 37 presented statistically different relative abundances in their specific terroir (Figure 8), while only 1 prokaryotic function and two fungi ecologies presented statistical differences. Assobio was the terroir with higher number of indicators taxa (14), being most of them low abundance taxa, with exception of *Paraburkholderia* species that were highly represented. Five of these species were exclusive to Assobio terroir samples while seven others were also present in other terroirs with different abundances (Figure S5). These indicator species belong to six different phyla (Bacteroidetes, Proteobacteria, Actinobacteria, Ascomycota, Basidiomycota, Firmicutes), being the Genus *Paraburkholderia* the only with two species. Assobio was also the only terroir with identified indicator function (dark iron oxidation) and fungi ecologies (“litter saprotroph” as primary lifestyle and “gills” Hymenium type). Twelve indicator species were identified for Margem terroir samples, six were exclusively present in this terroir (Figure S5). Margem indicator species belong to nine different phyla (Synergistetes, Actinobacteria, Thaumarchaeota, Verrucomicrobia, Ascomycota, Proteobacteria, Nitrospirae, Firmicutes, Deinococcus-Thermus), all with different Genus. Vinhas Velhas terroir samples revealed to have seven indicator species, with only one being exclusive to this terroir. Vinhas Velhas samples indicator species belonging to two phyla, Bacteroidetes and Actinobacteria, with six of these indicator species belong to the Genus *Mycobacterium*. Reserva terroir revealed the lower number of indicator species, four species belonging to tree different phyla, Actinobacteria, Ascomycota and Acidobacteria.

### 3.5. Grapevine Pathogens

Grapevine pathogens were also found in the terroir soil samples; their relative abundance was rather low. Despite this low abundance, the identification of grapevine pathogens is important when considering management strategies. *Plasmopara viticola*, the etiological agents of downy mildew, were identified in all terroirs while *Botrytis cinerea* (the etiological agent of gray mold), was not present in Margem and Vinhas Velhas (Table S4). *Erysiphe necator,* the etiological agent of powdery mildew, was only present in Vinhas Velhas terroirs (Table S4). Several pathogens associated with grapevine truck diseases (GTD) were found, namely: *Eutypa lata*, *Lasiodiplodia theobromae* and *Neofusicoccum parvum*. These were the Four pathogens that presented higher abundance, particularly in Margem. Other GTD associated pathogens were found, *Fomitiporia mediterranea*, present in all terroirs and *Botryosphaeria dothidea*, present in Margem terroirs only. Fungi associated with esca syndrome development, *Phaeomoniella chlamydospore* and *Phaeoacremonium minimum*, were also found with higher abundances in Margem terroirs (Table S4).

## 4. Discussion

Microbial communities associated with the vineyard play an important role in soil and plant productivity and fitness. Soil is the main microbiota reservoir and the specific (non-random) association between microorganisms and a particular geographical region reveals the potential applied impact of microbial terroirs [33]. The definition of microbial terroirs as well as the understanding of global patterns in the microbial community composition of specific vineyard soils may prompt the definition of adequate strategies (either agricultural or biotechnological) for productivity, disease resistance and wine sensorial traits. Also, information on the presence of grapevine pathogens may also pinpoint targets for monitoring throughout the crop season or enable the reduction of chemical treatments and definition of eradication strategies.

Previous studies of vineyard soil microbiome were conducted by focusing on bacteria and fungi communities through Illumina sequencing [33,34,35]. To the authors knowledge this is the first metagenomic study of terroirs soil microbiome utilizing the long-reads Oxford nanopores sequencing technique. This technique was selected based on its ability to sequence whole genome without the amplification bias (especially important for discrimination between different samples) and allowing the discrimination of closed related species. This study also allowed for the identification of indicator species and function specific for each terror. Previous study also identified species that contributed for soil distinction but at a global scale [33].

Reserva was the terroir with lower organic matter values, which is coherent with the lower abundance of wood decay fungi such as Agaricomycetes [36]. This is also reflected when analysing the fungi ecology guilds that, apart from exception of “Arbuscular mycorrhizal fungi”, and “other root-associated fungi”, showed lower relative abundances. On the other hand, this higher presence of arbuscular mycorrhizal may result from lower concentration of phosphate and nitrate present in Reserva soil, as a strategy for improved nutrient uptake by the plants (review by [37]). Considering indicator species, Reserva was the terroir where a lower number of indicator species were identified, however its indicator species present roles in nitrogen and phosphate cycles, as well as biomass degradation. This is the case of *Tetrasphaera* sp. HKS02, a genus *Tetrasphaera* well known for its denitrification activity and Phosphorus (P) uptake ( [38], reviewed in [39]). Moreover, *Sinomonas atrocyanea*, also identified as a Reserva terroir indicator species displays nitrate reduction and urease activities [40,41] as well as indole acetic acid production, and phosphate solubilization capacity [42]. These functions follow the same trend than Phosphate and nitrogen values found on this terroir.

Vinhas Velhas was the terroir most similar to Reserva, considering soil physic-chemical characteristics, namely lower pH values and nitrate concentration. Total and soluble inorganic phosphate concentrations were high in Vinhas Velhas, similarly to Margem. Even though some soil characteristics were mostly similar to Reserva, Vinhas Velhas presented a higher microbial abundance. Several taxa may relate to the soil properties, namely the lower abundance of Mucoromycota, which may associate with the lower pH and higher Phosphate concentration found [43], and the lower relative abundance of Betaproteobacteria, which may also be associated with the pH and lower nitrate concentration [44]. Of the eight Vinhas Velhas indicator species, six belong to the genus *Mycobacterium*. When analysing deeper this genus, from all Mycobacteria species found (genus *Mycobacterium*, *Mycolicibacterium*, *Mycolicibacter*, and *Mycobacteroides*), 70% of these presented higher read counts in the Vinhas Velhas terroirs them in other terroirs. Mycobacteria are ubiquitous bacteria present in a broad type of soils and environments; however high abundances of these bacteria are found in more acidic soils [45]. Although *Mycobacterium* are mainly studied as human and animal pathogens, previous studies indicate that the same species can act as root nodule endophytes, promoting soil nutrient turnover and/or being plant beneficial agents [46,47,48,49,50,51].

Assobio and Margem terroirs revealed to be the most similar, when considering the studied parameters. Only their inorganic phosphate concentration was significantly different, with Margem presenting a ten times higher concentration. These were also the terroirs with the highest relative abundance in most of the analysed taxa. Assobio and Margem showed higher aerobic nitrite oxidation and nitrification function, which may be associated with the higher nitrate values found, being this a highly important microbial function while oxidizing ammonia and nitrite to nitrate, the preferred N uptake form for plants [52]. At the class level, Margem terroir presented the highest abundance of Deltaproteobacteria. This class is characterized by sulphate and sulphur reduction bacteria [53,54,55], dissimilative iron reducers [56] and bacterial predators [57]. Margem soil’s higher nitrate concentration may impact nitrogen fixation bacteria abundance since high reactive nitrogen (nitrate, ammonium, or organic nitrogen) concentrations may inhibit the nitrogenase complex [58,59] and decrease the adaptative advantage of the diazotrophic function. Interestingly, one of the Margem indicator species belongs to a genus associated to nitrogen fixation, *Mesorhizobium* (reviewed in [60], [61,62]). Other indicator species for Margem, are also involved in the nitrogen metabolic cycle (*Nitrospira moscoviensis* [63]; *Neisseria cinerea* [64]; Candidatus *Nitrososphaera gargensis* [65]). The presence of microorganisms able to oxidize ammonia and nitrite, as well as nitrogen fixation may reflect the high nitrate concentration present is this terroir.

Assobio is the terroir that presents the highest number of indicator functions and fungi ecological guilds with significantly higher abundances when compared to other terroirs. Some Assobio indicator species may present biocontrol roles (*Collimonas fungivorans* [66,67]), other less explored/known functions (*Suhomyces canberraensis* [68], *Flaviflexus ciconiae* [69], *Hygrocybe conica* [70]), and a high number of plant growth promoting species (*Paraburkholderia graminis* [71,72], *Paraburkholderia phytofirmans* [73,74], *Sphingobacterium multivorum* [75], *Oidiodendron maius* [76], *Enterobacter* sp. [77,78], *Talaromyces islandicus* [79]). These plant-growth promoting microbes are the main group of indicator species found in Assobio terroir. Of those, *P. graminis* has been shown to increase the nitrogen content of plants through an abundance increase of high-affinity nitrate transporter NAR2 and its activator, as well as increase in ammonium-inducible transporter [72]. *Paraburkholderia phytofirmans* colonised tomato plants showed an increase in photosynthesis and photosystem II activity even in higher temperatures [73], and *P. phytofirmans* volatile organic compound conferred tolerance to salinity and increased Arabidopsis growth [74]. *Sphingobacterium multivorum* growth promoting mechanisms are mostly indole-3-acetic acid (IAA) and siderophore production, 1-aminocyclopropane-1-carboxylic acid (ACC) deaminase activity and phosphate solubilization [75]. *Oidiodendron maius* is an ericoid mycorrhizal fungus, with plant growth promoting activity through increased nitrogen uptake [76]. *Enterobacter* spp. are able to promote plant growth by aiding nitrogen fixation [77], IAA production [78] and by heavy metal removal from soil through siderophore production [77]. *Talaromyces islandicus* displays phosphorus solubilization activity improving maise growth and phosphorus uptake [79]. When looking at the potential indicator functions, dark iron oxidation was appointed to Assobio, a terroir with more neutral pH and high concentration of nitrate, which may explain this indicator. Some of the species identified as being able to perform iron oxidation are neutrophilic, aerobic iron-oxidizing proteobacteria or neutrophilic iron-oxidizing proteobacteria, all of which use nitrate as respiratory substrates [80]. Also, in Assobio terroir, two species of yeast were identified as indicator species. Not much is known about the species *Kazachstania kunashirensis*, however other species from this genus were previously reported as conferring positive aroma attributes to wine in the presence of *Saccharomyces cerevisiae* [81,82]. *Suhomyces* species seem to be able to convert glucose and trehalose to alcohol by fermentation, thus aiding the wine-making process [68].

In our study, several grapevine pathogens were identified, Margem soil may constitute a reservoir for grapevine trunk diseases (*Eutypa lata, Lasiodiplodia theobromae, Neofusicoccum parvum* and *Botryosphaeria dothidea*) and esca syndrome pathogens (*Phaeomoniella chlamydospore* and *Phaeoacremonium minimum*) based on pathogens abundance and higher mortality rate of grapevine in this terroir in the last years, further corroborating that soil may be an important source of GTD inoculum [13]. Additionally, an uncharacterized *Fusarium* sp. S18/39 species was found to be an indicator species for Margem terroir soils. Although many *Fusarium* species are grapevine pathogens [83,84], some may also have a biocontrol action against other diseases [85,86,87], thus further studies on this particular species are needed to enlighten its role within grapevine interaction.

Overall, the studied microbiomes reflect the different terroirs, with the possibility to identity indicator species and function/ecologies to each terroir. In the future, taking into account that the soil is considered a plant reservoir of microorganisms, a microbiome study of the must will be interesting to better understand the microorganisms that reflect the differences in wine quality of each terroir.

## 5. Conclusions

We have used a long-read sequencing approach to trace and define “microbial terroirs” at an organic vineyard at the Douro region. Our results demonstrated that the soil microbiome constitutes a terroir signature. Each terroir was also associated with signature functions, ecologies, and indicator species, suggesting that microbiome analysis is a viable method to distinguish terroirs. This function and indicator species are not only important for terroirs distinguish but also for plant development and potential wine production, reflecting the variety and quality of wine produced in this region. This result may aid the definition of adequate viticulture and oenological practices.

## Figures and Tables

**Table 1:** Terroir geographical and soil characteristics. In all terroirs the grapevines were conducted in the vertical.

Terroir	Altitude (m)	Sun Exposure	Types of Plantations	Soil Composition	Year of Plantation
Margem	140–170	SouthWest	Vertical	schist blocks and pebbles rolled schist	1980–1987
Vinhas Velhas	260–290	South-East	Vertical	schist	1947
Assobio	231–308	North	Vertical	mica-schist	1987
Reserva	150–280	SouthWest	Vertical	mica-schist	1980–1987

**Table 2:** Mean and standard deviation values for each of the soil physicochemical parameters analysed for Assobio, Vinhas Velhas, Margem and Reserva. Different letters indicate statistically significant differences between the terroirs, considering *p* < 0.05 (n = 5).

	Assobio	Vinhas Velhas	Margem	Reserva
pH	6.15 ± 0.06 ^a^	5.69± 0.2 ^b^	6.15 ± 0.21 ^a^	5.98 ± 0.15 ^ab^
OM (%)	5.54 ± 0.12 ^a^	5.11 ± 0.47 ^a^	4.98 ± 0.86 ^a^	3.82 ± 0.18 ^b^
Soil water content (%)	1.65 ± 0.55 ^a^	1.16 ± 0.51 ^a^	2.08 ± 0.63 ^a^	1.77 ± 0.28 ^a^
Inorganic soluble PO_4_^−^ (µg/g soil)	2.66 ± 1.62 ^c^	20.3 ± 0.46 ^ab^	27.4 ± 13.46 ^a^	9.07 ± 1.92 ^bc^
Total phosphate (mg/g soil)	0.18 ± 0.03 ^a^	0.61 ± 0.43 ^a^	0.30 ± 0.11 ^a^	0.01 ± 0.01 ^b^
Nitrate (μg/g soil)	10.76 ± 4.62 ^a^	3.67 ± 0.67 ^b^	17.85 ± 9.38 ^a^	3.25 ± 1.27 ^b^

## Data Availability

The data presented in this study are available on request from the corresponding author.

## Supplementary Materials

The following supporting information can be downloaded at: https://www.mdpi.com/article/10.3390/microorganisms11030672/s1, Figure S1: Rarefaction curve showing number of observed taxa in each terroir sample; Figure S2: Relative abundance of kingdom (A), Archaea phyla (B), Eukaryota phyla (C), Virus phyla (D) and Bacteria phyla (E) in Quinta dos Murças soil samples. Relative abundance for phyla is calculated within each kingdom group. Individual data points from libraries from different terroirs are shown to give an overall perspective on data variability; Figure S3: Terroir associated Prokaryotes function predicted by FAPROTAX database. Relative Abundance (0–1) hierarchical clustering based on squared Euclidean distance and complete linkage method; Figure S4: Terroir associated Fungal functions predicted by FungalTraits database. Relative Abundance (0–1) hierarchical clustering based on squared Euclidean distance and complete linkage method; Figure S5: Venn diagram representing the terroir where the 37 identified potential indicator species can be found; Table S1: Prokaryotic functions based on database FAPROTAX; Table S2: Fungi ecologies based on database FungalTraits; Table S3: Number different taxonomic level present in each Terroir.; Table S4: Abundance of detected pathogens in Quinta dos Murças terroirs soils.
