# Supplementary material for: A New Perspective for Vineyard Terroir Identity: Looking for Microbial Indicator Species by Long Read Nanopore Sequencing
Source: Microorganisms. 2023 Mar 6;11(3):672. doi: 10.3390/microorganisms11030672 (PMC10054463; doi:10.3390/microorganisms11030672)
Supplement: Supplementary file 1 [file microorganisms-11-00672-s001.zip › Supplementary materials.zip!/Supplementary materials.pdf]

A new perspective for vineyard terroirs identity: looking for microbial indicator species by Long Read Nanopore Sequencing

Ana Cruz-Silva [1,2], Gonçalo Laureano [1,2], Marcelo Pereira [1], Ricardo Dias [1,3], José Moreira da Silva [4], Nuno Oliveira [5], Catarina Gouveia [1,2], Cristina Cruz [3,6], Margarida Gama-Carvalho [1,7], Fiammetta Alagna [8], Bernardo Duarte [3,9] and Andreia Figueiredo [1,2,3,*]

[1] Biosystems & Integrative Sciences Institute (BioISI), Faculdade de Ciências da Universidade de Lisboa, Lisboa, Portugal. amcsilva@fc.ul.pt (A.C.-S.); gmlaureano@fc.ul.pt (G.L.); mlpereira@fc.ul.pt (M.P.); rpdias@fc.ul.pt (R.D.); cagouveia@fc.ul.pt (C.G.); mhcarvalho@fc.ul.pt (M.G-C.)

[2] Grapevine Pathogen Systems Lab, BioISI Faculdade de Ciências da Universidade de Lisboa, Lisboa, Portugal.

[3] Departamento de Biologia Vegetal, Faculdade de Ciências da Universidade de Lisboa, Campo Grande, 1749-016, Lisboa, Portugal. ccruz@fc.ul.pt (C.C.); baduarte@fc.ul.pt (B.D.)

[4] Quinta dos Murças, Esporão Company, Douro, Portugal. jose.luis@esporao.com (J.M.S)

[5] NBI – Natural Business Intelligence, Regia Douro Park, 5000-033 Vila Real, Portugal. nuno.oliveira@nbi.pt (N.O.)

[6] cE3c - Center for Ecology, Evolution and Environmental Changes & CHANGE - Global Change and Sustainability Institute, Faculdade de Ciências da Universidade de Lisboa, Campo Grande, 1749-016, Lisboa, Portugal.

[7] Departamento de Química e Bioquímica, Faculdade de Ciências da Universidade de Lisboa, Campo Grande, 1749-016, Lisboa, Portugal.

[8] Energy Technologies and Renewable Sources Department, National Agency for New Technologies, Energy and Sustainable Economic Development (ENEA), Trisaia Research Centre, Rotondella, Matera, Italy. fiammetta.alagna@enea.it (F.A.)

[9] MARE – Marine and Environmental Sciences Centre & ARNET – Aquatic Research Infrastructure Network Associate Laboratory, Faculty of Sciences of the University of Lisbon, Campo Grande 1749-016 Lisbon, Portugal.

* Correspondence: aafigueiredo@fc.ul.pt

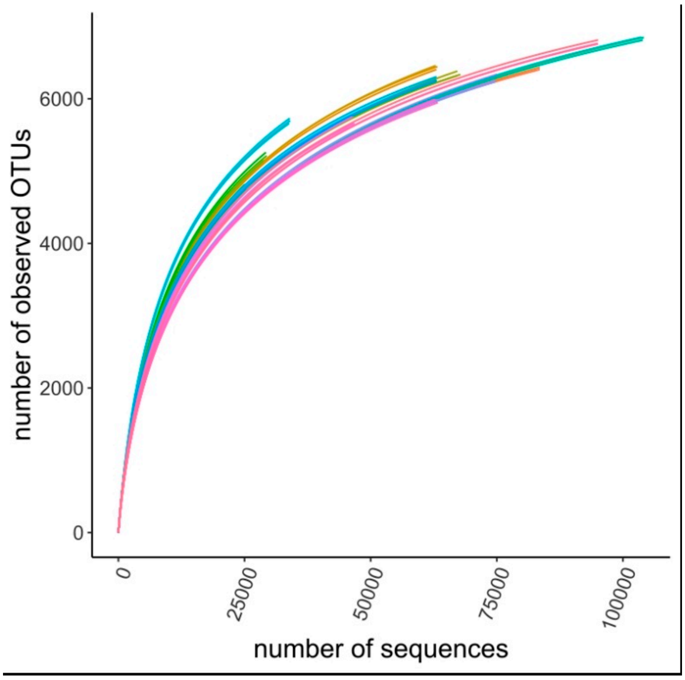

Figure S1: Rarefaction curve showing number of observed taxa in each terroir sample.

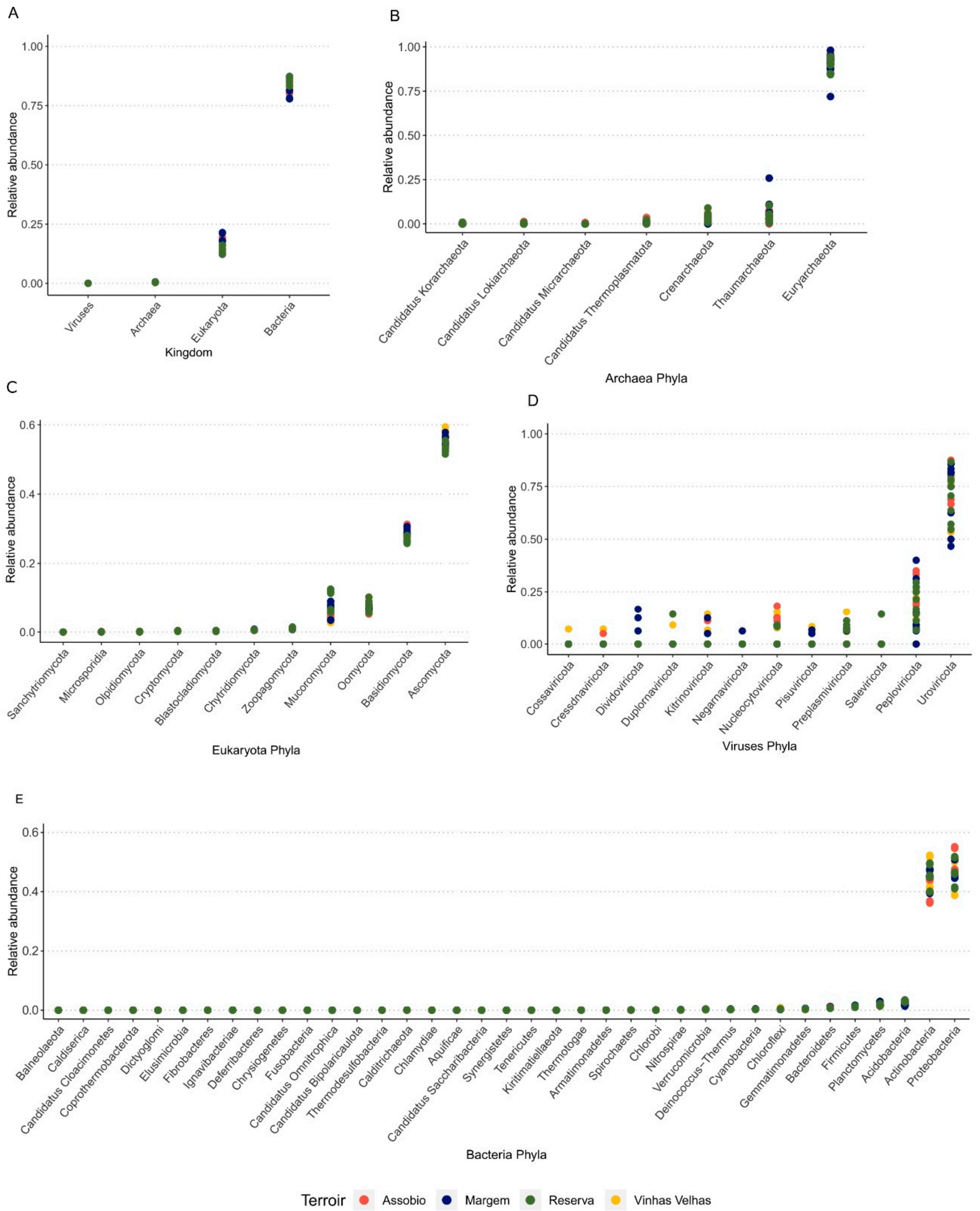

Figure S2: Relative abundance of kingdom (**A**), Archaea phyla (**B**), Eukaryota phyla (**C**), Virus phyla (**D**) and Bacteria phyla (**E**) in Quinta dos Murças soil samples. Relative abundance for phyla is calculated within each kingdom group. Individual data points from libraries from different terroirs are shown to give an overall perspective on data variability.

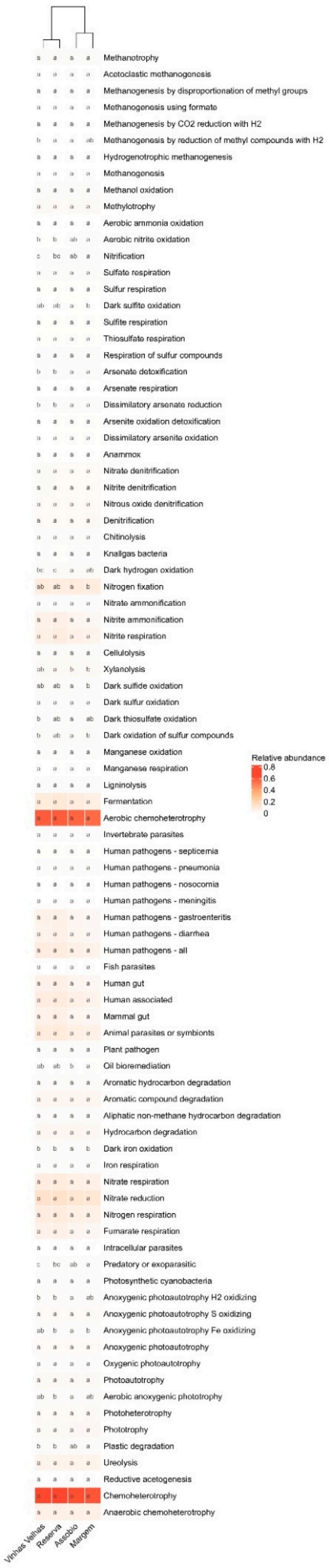

Figure S3: Terroir associated Prokaryotes function predicted by FAPROTAX database. Relative Abundance (0-1) hierarchical clustering based on squared Euclidean distance and complete linkage method.

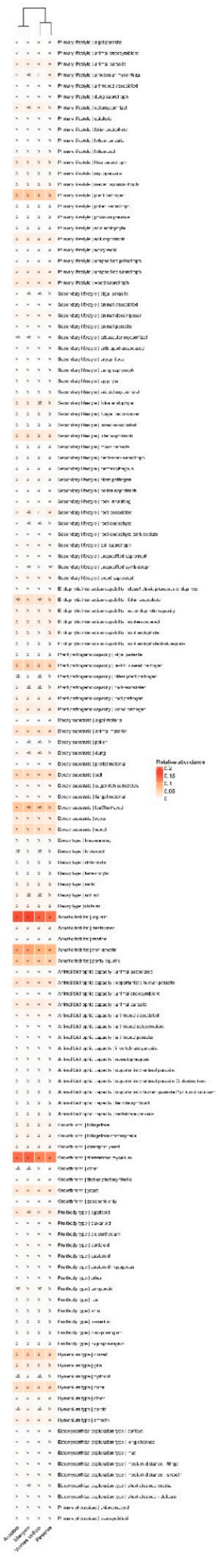

Figure S4: Terroir associated Fungal functions predicted by FungalTraits database. Relative Abundance (0-1) hierarchical clustering based on squared Euclidean distance and complete linkage method.

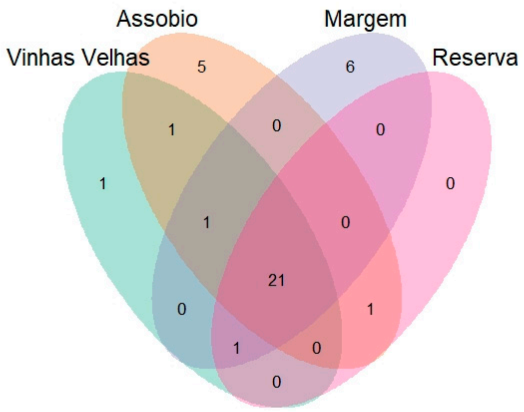

Figure S5: Venn diagram representing the terroir where the 37 identified potential indicator species can be found.

Table S3: Number different taxonomic level present in each Terroir.

	Number of different taxonomic levels					
	Phylum	Class	Order	Family	Genus	Species
Assobio	57	135	331	798	2 355	7 300
Margem	60	138	335	798	2 366	7 301
Reserva	58	134	330	789	2 325	7 131
Vinhas Velhas	61	141	339	798	2 343	7 238
Total	67	149	351	841	2 633	8 558

Table S4: Abundance of detected pathogens in Quinta dos Murças terroirs soils.

	Vinhas Velhas	Assobio	Margem	Reserva
Plasmopara viticola	$4.44x10^{-5} \pm 4.21x10^{-5}$	$2.22x10^{-5} \pm 2.90x10^{-5}$	$5.77x10^{-5} \pm 5.32x10^{-5}$	$4.44x10^{-5} \pm 4.44x10^{-5}$
Botrytis cinerea	0	$2.22x10^{-5} \pm 4.05x10^{-5}$	0	$2.22x10^{-5} \pm 2.22x10^{-5}$
Erysiphe necator	$1.33x10^{-5} \pm 2.00\ x10^{-5}$	0	0	0
Phaeomoniella chlamydospora	$1.33x10^{-5} \pm 2.82x10^{-5}$	$3.99x10^{-5} \pm 3.46x10^{-5}$	$4.88x10^{-5} \pm 5.91x10^{-5}$	$3.10x10^{-5} \pm 3.10x10^{-5}$
Phaeoacremonium minimum	$5.77x10^{-5} \pm 4.05x10^{-5}$	$8.43x10^{-5} \pm 5.80x10^{-5}$	$5.59x10^{-4} \pm 6.39x10^{-4}$	$4.44x10^{-5} \pm 4.44x10^{-5}$
Eutypa lata	$1.15x10^{-4} \pm 7.32x10^{-5}$	$6.65x10^{-5} \pm 6.91x10^{-5}$	$1.38x10^{-4} \pm 1.18x10^{-4}$	$1.02x10^{-4} \pm 1.02\ x10^{-4}$
Lasiodiplodia theobromae	$7.54x10^{-5} \pm 4.66x10^{-5}$	$7.98x10^{-5} \pm 6.62x10^{-5}$	$1.29x10^{-4} \pm 2.66x10^{-5}$	$3.99x10^{-5} \pm 3.99x10^{-5}$
Neofusicoccum parvum	$3.55x10^{-5} \pm 3.70x10^{-5}$	$6.21x10^{-5} \pm 4.51x10^{-5}$	$9.31x10^{-5} \pm 4.89x10^{-5}$	$4.44x10^{-5} \pm 4.44x10^{-5}$
Botryosphaeria dothidea	0	0	$8.87x10^{-6} \pm 1.76x10^{-5}$	0
Fomitiporia mediterranea	$3.1x10^{-5} \pm 4.36x10^{-5}$	$1.77x10^{-5} \pm 4.05x10^{-5}$	$2.22x10^{-5} \pm 4.51x10^{-5}$	$1.77x10^{-5} \pm 1.77\ x10^{-5}$
